# MiR-17 and miR-19 cooperatively promote skeletal muscle cell differentiation

**DOI:** 10.1007/s00018-019-03165-7

**Published:** 2019-06-18

**Authors:** Delin Kong, Mei He, Lin Yang, Rongtao Zhou, Yun-Qin Yan, Yang Liang, Chun-Bo Teng

**Affiliations:** 1 College of Life Science, Northeast Forestry University, Harbin, 150040 China; 2 The Laboratory of Cell and Developmental Biology, Northeast Agricultural University, Harbin, China

**Keywords:** microRNA, miR-17–92, Skeletal myogenesis, Myogenic differentiation, Muscle regeneration

## Abstract

Skeletal myogenesis is a highly coordinated process that involves cell proliferation, differentiation and fusion controlled by a complex gene regulatory network. The microRNA gene cluster miR-17–92 has been shown to be related to this process; however, the exact role of each cluster member remains unclear. Here, we show that miR-17 and miR-20a could effectively promote the differentiation of both C2C12 myoblasts and primary bovine satellite cells. In contrast, miR-18a might play a negative role in C2C12 cell differentiation, while miR-19 and miR-92a had little influence. Transcriptome and target analyses revealed that miR-17 could act on *Ccnd2*, *Jak1* and *Rhoc* genes that are critical for cell proliferation and/or fusion. Notably, the addition of miR-19 could reverse the lethal effect of miR-17 and could thus facilitate the maturation of myotubes. Furthermore, by co-injecting the lentiviral shRNAs of miR-17 and miR-19 into mouse tibialis anterior muscles, we demonstrated the wound healing abilities of the two miRNAs. Our findings indicate that in combination with miR-19, miR-17 is a potent inducer of skeletal muscle differentiation.

**Electronic supplementary material:**

The online version of this article (10.1007/s00018-019-03165-7) contains supplementary material, which is available to authorized users.

## Introduction

In vertebrates, skeletal myogenesis is a highly coordinated process that is fundamental to the development, growth and regeneration of skeletal muscle. Particularly, in response to exercise or damage, skeletal muscle can robustly regenerate owing to the residence of multipotent satellite cells [1]. Upon activation, satellite cells exit the quiescent state to proliferate and differentiate into myoblasts, which then further differentiate and fuse into multinucleated myotubes [2]. The multistep myogenic process is tightly controlled by a complex gene regulatory network. A group of bHLH (basic helix–loop–helix) transcription factors is located at the core node in this network, namely MRFs (myogenic regulatory factors), including MYF5, MYOD, MYOG (myogenin), and MRF4 [3, 4]. Many miRNAs (microRNAs), small non-coding RNA molecules that target mRNAs to fine-tune gene expression, are also important components of this network [5–8].

The highly conserved miR-17–92 cluster is one of the most investigated miRNA clusters. It consists of six miRNAs as follows: miR-17, -18a, -19a, -20a, -19b-1, and -92a-1 (Fig. S1a). In the mature form, miR-19a and -19b share exactly the same sequence, while miR-17 and -20a only differ in two nucleotides [9, 10]. Although co-transcribed initially, individual members of this cluster may have cooperative or opposing effects depending on the context. The miR-17–92 cluster was first found to be an oncomir in malignant B cell lymphoma [11]. Subsequently, miR-19a and -19b were identified to be the key oncogenic ones by targeting PTEN (phosphate and tensin homologue), thus increasing the PI3K–AKT signalling pathway for the maintenance and survival of B-lymphoma; however, miR-17 did not contribute significantly to the malignant transformation of B-lymphoma [12, 13]. By contrast, in retinoblastoma, miR-17 and -20a became the key oncogenic ones, largely due to the repression of P21 (cyclin-dependent kinase inhibitor) and the TGF-beta pathway components [14, 15]. It is increasingly clear that the role of each miR-17–92 member varies in a context-dependent manner. Most studies characterised miR-17 as a pro-proliferative miRNA contributing to tumourigenesis in various cell types [16, 17]. However, the anti-proliferative effect of miR-17 has also been noticed in specific contexts (e.g., breast cancer cells) [18–20].

Notably, an inconsistency has arisen with respect to the roles of the miR-17–92 cluster in skeletal myogenesis. Liu et al. [21] demonstrated that by binding to the miR-17–92 promoter in proliferating C2C12 myoblasts, exogenous MYOG could upregulate miR-20a to force cell cycle exit and stimulate differentiation. Luo et al. [22] also showed that the chemosynthetic mimics of miR-20a-5p and miR-20b-5p inhibited proliferation and promoted differentiation in QM-7 (quail muscle clone 7) cells, which involved an auto-regulatory feedback loop between E2F1 and miR-20a/b. On the contrary, Qiu et al. [23] reported that in C2C12 cells, miR-17, -20a or -92a enhanced proliferation and repressed differentiation by targeting ENH1 (actin-associated protein enigma homologue 1), thus increasing the nuclear accumulation of ID1 (inhibitor of differentiation 1) to block MRFs.

Therefore, to better understand the roles of the miR-17–92 cluster in muscle differentiation, we transfected the mimic of each cluster member into C2C12 cells and identified miR-17 and miR-20a, but not miR-18a, miR-19 or miR-92a, to be potent inducers of muscle differentiation. Nevertheless, miR-18a might be a repressor, as treating cells with its specific inhibitor facilitated myogenic differentiation. Transcriptome and target analyses revealed that the pro-differentiation ability of miR-17 was achieved in part by inhibiting the critical targets *Ccnd2* (cyclin D2), *Jak1* (Janus kinase 1) and *Rhoc* (ras homologue family member C). Notably, miR-19 could reverse the phenomenon of cell death caused by miR-17, and the simultaneous administration of both could significantly promote the differentiation of primary bovine skeletal muscle-derived satellite cells (MDSCs) and the repair of mouse tibialis anterior muscles. Our study not only revealed the mechanism by which miR-17 promotes skeletal muscle differentiation but also provided a potential strategy for meat production increase and muscle disease therapy.

## Results

### Different roles of the miR-17–92 cluster members in muscle differentiation

**Fig. 1:**
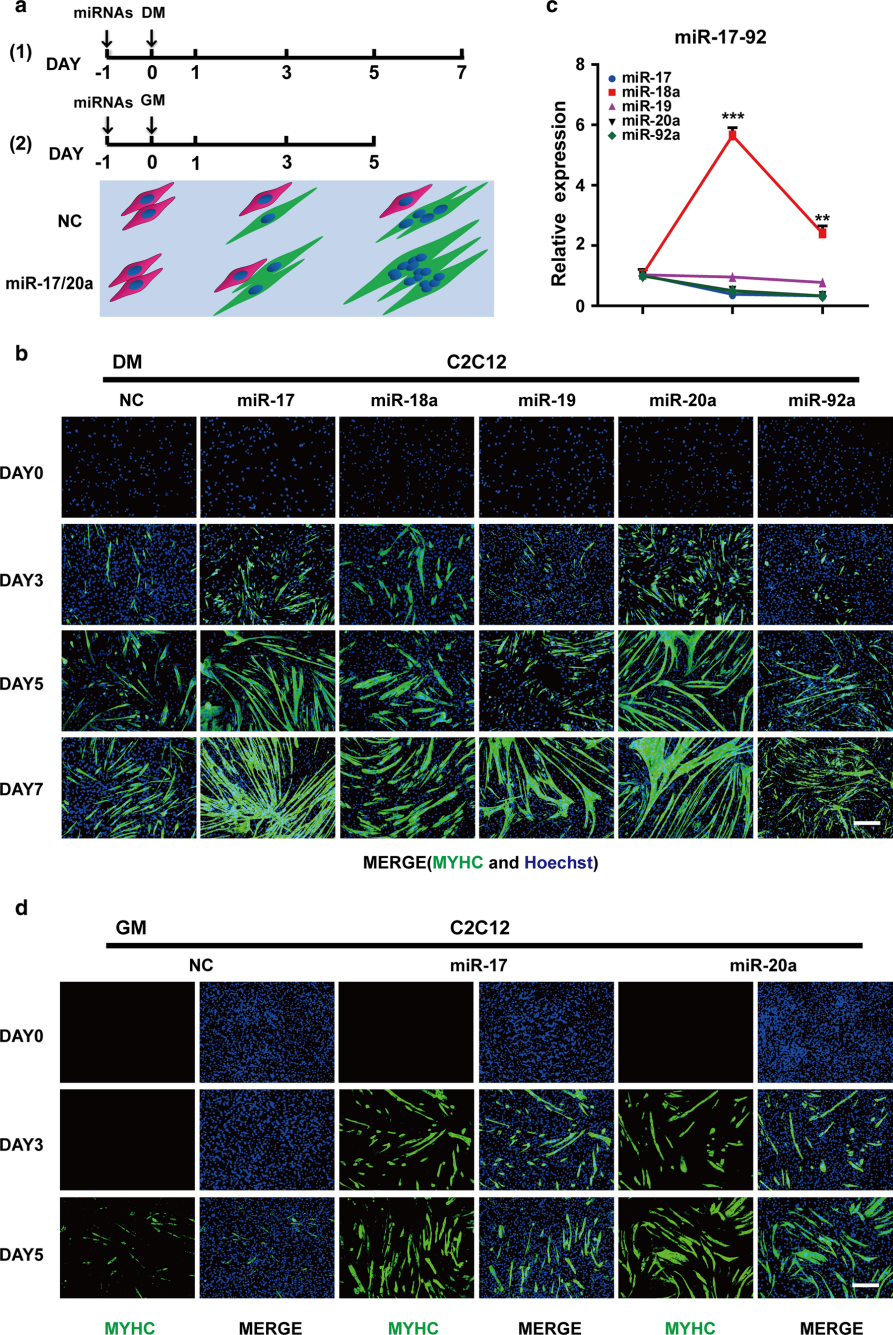
Different roles of the miR-17–92 cluster members in muscle differentiation. **a** Two strategies for the muscle differentiation assay. At 24 h after the transfection with the miRNA mimics or the NC (negative control, scrambled sequence), the cells were cultured in DM (differentiation medium) or GM (growth medium), and then examined on the indicated days. The pattern diagram represents the process of myogenic differentiation, with the myoblasts in red, the myotubes in green and the nuclei in blue. **b** MYHC immunostaining of C2C12 cells transfected with each miRNA mimic at the indicated time points during DM-induced differentiation. Among the miR-17–92 cluster members, miR-17 and miR-20a, but not the other three miRNAs, could advance the myogenic programme since day 3 (scale bar = 100 μm). **c** The endogenous expression patterns of the miR-17–92 cluster members during normal C2C12 cell differentiation. The levels of mature miRNAs were detected by qRT-PCR on days 1, 3 and 7. The relative (miRNA/U6) levels on day 1 were all set to 1.0. All members were downregulated, except miR-18 (mean ± SEM, **P < 0.01, ***P < 0.001). **d** miR-17 and miR-20a promoted C2C12 cell differentiation in GM (scale bar = 100 μm)

To analyse the effects of the miR-17–92 cluster on muscle differentiation, C2C12 myoblasts were transfected with each of the five miRNA mimics (miR-17-5p, miR-20a-5p, miR-19a-3p/miR-19b-3p, miR-18a-5p and miR-92a-3p) and then cultured in DM (differentiation medium) (Fig. 1a). Compared with that of the NC (negative control, scrambled sequence) cells, the myogenic programme was advanced in the cells carrying either the miR-17 or miR-20a mimic, with higher MYHC (myosin heavy chain) expression starting on day 3 and more myotube formation starting on day 5. Notably, most cells had already fused into long and multinucleated fibres on day 7. In contrast, the mimics of miR-18a, miR-19 and miR-92a had little influence on C2C12 cell differentiation (Figs. 1b, S1c).

Intriguingly, the expression levels of the miR-17–92 cluster members were all decreased during the normal differentiation of C2C12 cells, except for that of miR-18a, which increased during the early stages and decreased later (Fig. 1c). Its abundance was also much higher than that of the others all along (Fig. S1b). However, the exogenous addition of miR-18a had little pro-differentiation effects (Fig. 1b). Thus, we aimed to test whether the opposite conditions would work. Indeed, after the treatment with the miR-18a inhibitor, more cells expressed MYHC on day 3, and a larger bundle of myotubes was formed on day 7. By contrast, the miR-19 inhibitor did not show any apparent effects on myogenic differentiation (Fig. S1d, e), nor did the miR-17 inhibitor (data not shown).

Then, to further examine the pro-differentiation abilities of miR-17 and miR-20a, transfected cells were cultured in GM (growth medium) that is not conductive to C2C12 cell differentiation (Fig. 1a). Surprisingly, miR-17 and miR-20a still exhibited strong effects (Figs. 1d, S1f). After 48 h of incubation in GM, the two mimics were able to induce the expression of *Myh3* (myosin heavy chain 3) at concentrations as low as 2 nM. When their concentrations reached 50 nM, *Myh3* transcripts were significantly increased (Fig. S1g). For miR-19, although its level was also elevated upon transfection, there was no significant difference in the level of *Myh3* (Fig. S1g). Notably, miR-17 and miR-20a also accelerated the differentiation process of primary bovine MDSCs in DM (Fig. S1h, i), as was confirmed by the upregulated transcription of *MYH3*, *MYOD1* and *MYOG* (Fig. S1j).

### Transcriptomic changes induced by miR-17 or miR-20a

The knockdown of *Ago2* (argonaute 2) or *Gw182*, the two key proteins of RISC (RNA-induced silencing complex), indicated that miR-17 and miR-20a promoted C2C12 cell differentiation via the classical RISC degradation pathway (Fig. S2). Then, to view the changes in gene expression profile induced by miR-17 or miR-20a to accelerate the myogenic programme, C2C12 cells were transfected with the appropriate mimic for 48 h in GM and were harvested for RNA-seq analysis.

**Fig. 2:**
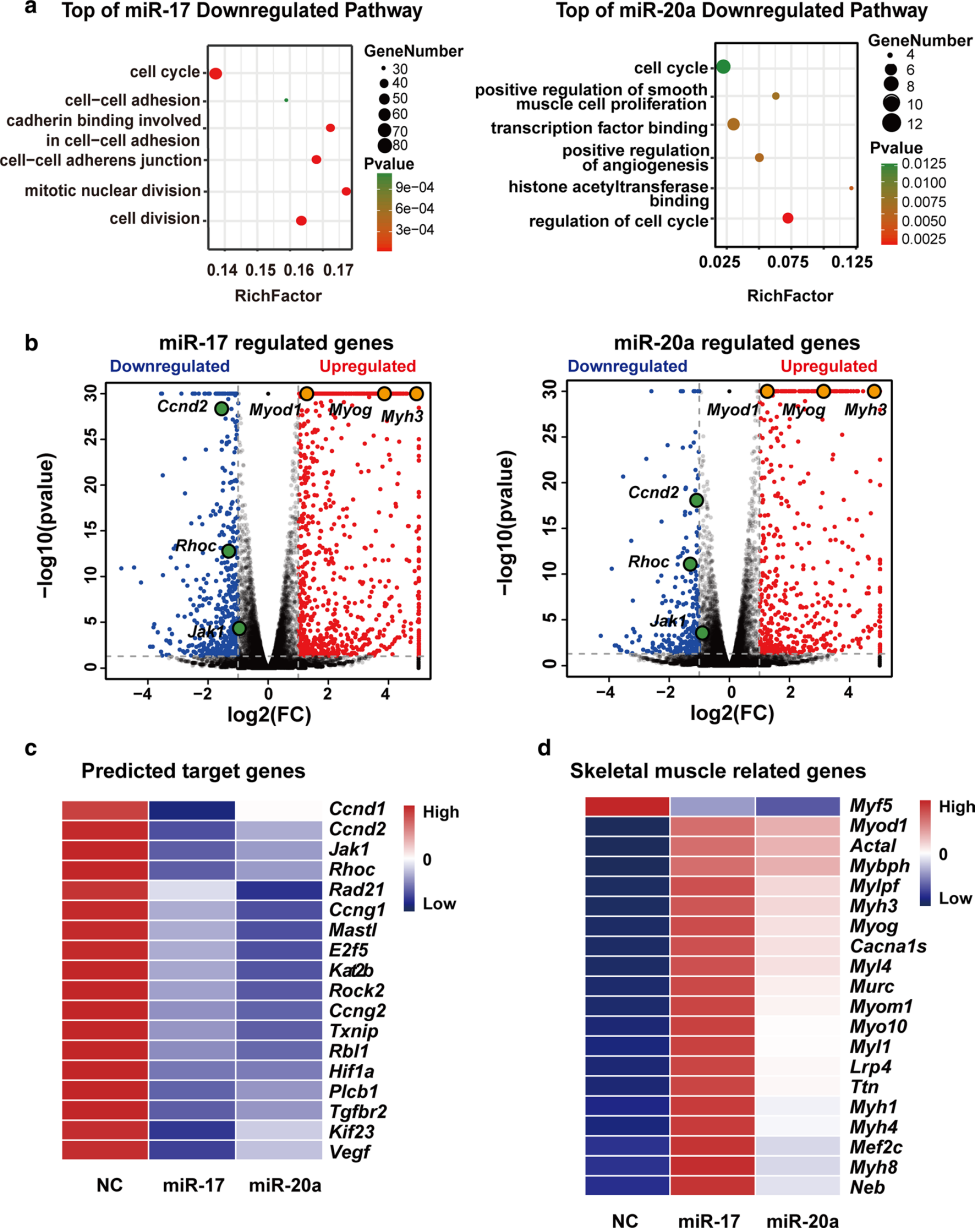
Transcriptomic changes induced by miR-17 or miR-20a. **a** The six top pathways downregulated by miR-17 or miR-20a according to GO (Gene Ontology) enrichment. RichFactor is equal to the downregulated gene number divided by the total gene number in the pathway. **b** Volcano plots of − log10 (adjusted *P* value) vs. log2 (fold change, FC) of all differential genes upon miR-17 or miR-20a treatment. The threshold (*P* = 0.05) is indicated with a dashed line. *Ccnd2*, *Jak1* and *Rhoc* are the key downregulated targets, while *Myh3*, *Myod1* and *Myog* are labelled as the major myogenic markers. **c** A heatmap of the Targetscan database’s representative predicted targets of miR-17 and miR-20a that were downregulated by their mimics according to the RNA-seq data. **d** A heatmap of the representative skeletal muscle-related genes that were differentially expressed upon miR-17 or miR-20a treatment. Notably, *Myf5* was decreased by both miRNAs, and miR-17 induced higher expression levels of the other genes

The functional categories of genes that were downregulated by miR-17 or miR-20a suggested a particular enrichment of cell cycle-related genes (Fig. 2a). Notably, *Ccnd2*, *Jak1* and *Rhoc* were among the genes significantly downregulated (Fig. 2b). Actually, the three genes, together with some other downregulated genes associated with cell proliferation (Fig. 2c), were predicted to be the common targets of miR-17 and miR-20a by all three databases (TargetScan, MicroRNA and MiRDB) due to their identical seed sequences.

It should also be noted that the level of *Myf5*, which is essential for satellite cell/myoblast proliferation, was reduced in both samples; this is in contrast to the levels of *Myod1* and *Myog*, which are required for myotube formation (Fig. 2d). Meanwhile, there was little change in *Mrf4* expression (data not shown). Moreover, miR-17 seemed to have a stronger ability than that of miR-20a to induce the differentiation-related genes (Fig. 2d). Thus, we focused on miR-17 in the subsequent experiments.

### *Ccnd2*, *Jak1* and *Rhoc* were directly targeted by miR-17 to promote C2C12 cell differentiation

**Fig. 3:**
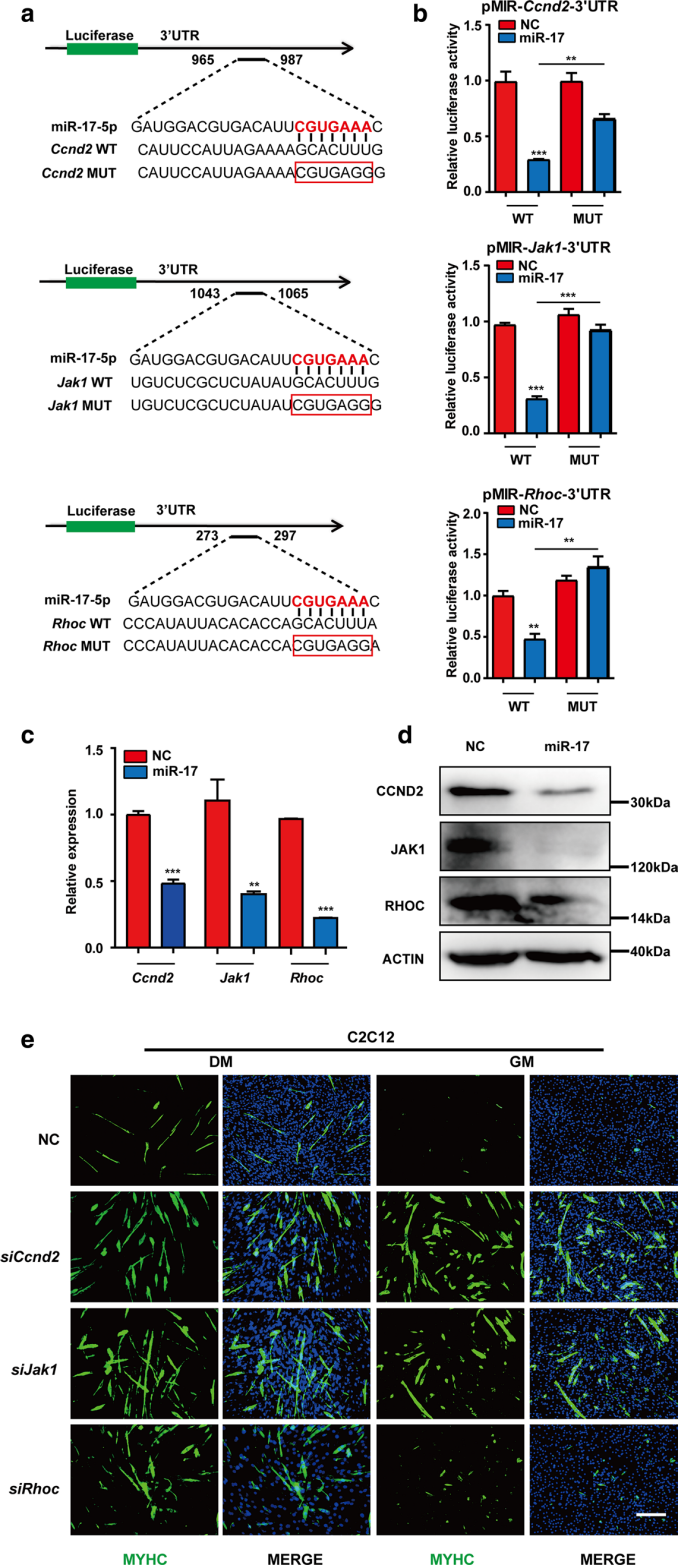
*Ccnd2*, *Jak1* and *Rhoc* were directly targeted by miR-17 in promoting C2C12 cell differentiation. **a** A sketch map of the predicted miR-17-binding sites in the 3′UTR regions of the *Ccnd2*, *Jak1* and *Rhoc* mRNAs. The seed region of miR-17-5p is highlighted in red and base paired with the wide-type sequence (WT) of each mRNA. The corresponding mutant sequence (MUT) is indicated with a red box. **b** A dual-luciferase reporter assay. The 3′UTR fragments (WT and MUT) of *Ccnd2*, *Jak1* and *Rhoc* were cloned into the pMIR vector and then co-transfected with the miR-17 mimic or the NC (negative control, scrambled sequence). The relative Firefly/Renilla luciferase activity in the WT and NC group was set to 1.0 (mean + SEM, **P < 0.01, ***P < 0.001). **c** The mRNA levels of *Ccnd2*, *Jak1* and *Rhoc* were decreased by miR-17. The relative (*mRNA*/*Gapdh*) levels in the NC were set to 1.0 (mean + SEM, ***P < 0.001). **d** The protein levels of CCND2, JAK1 and RHOC were decreased by miR-17 according to the western result. ACTIN was used as the internal control. **e** MYHC immunostaining of C2C12 cells treated with the siRNAs of *Ccnd2*, *Jak1* and *Rhoc*. On day 3, all siRNAs advanced the myogenic programme in DM (differentiation medium). Notably, *siCcnd2* and *siJak1* also worked in GM (growth medium) (scale bar = 100 μm)

To examine whether *Ccnd2*, *Jak1* and *Rhoc* were direct targets of miR-17, the dual-luciferase reporter system was employed. The 3′UTR fragments of the three genes containing putative miR-17 sites were cloned and inserted downstream of the firefly luciferase-coding region (Fig. 3a). The wild-type 3′UTR of these genes conferred significant repression (50–70%) on the firefly/Renilla ratio upon miR-17 co-transfection, which was reversed to different extent by site-specific mutagenesis (Fig. 3b).

Further, qPCR and western blot analyses demonstrated that both mRNA and protein levels of *Ccnd2*, *Jak1* and *Rhoc* were reduced by miR-17 (Figs. 3c, d, S3a). In fact, the suppression of the three genes by their siRNAs (Fig. S3b, c) could promote C2C12 cell differentiation after the incubation in DM for 3 days, respectively. Notably, *siCcnd2* and *siJak1* were also successful in GM (Figs. 3e, S3d). These results indicated that the effects of miR-17 could be achieved in part by directly targeting *Ccnd2* and *Jak1* that are well known for maintaining cell proliferation, as well as *Rhoc* that is more involved in suppressing cell motility and regulating cell fusion.

### miR-19 complemented miR-17 in promoting muscle differentiation

**Fig. 4:**
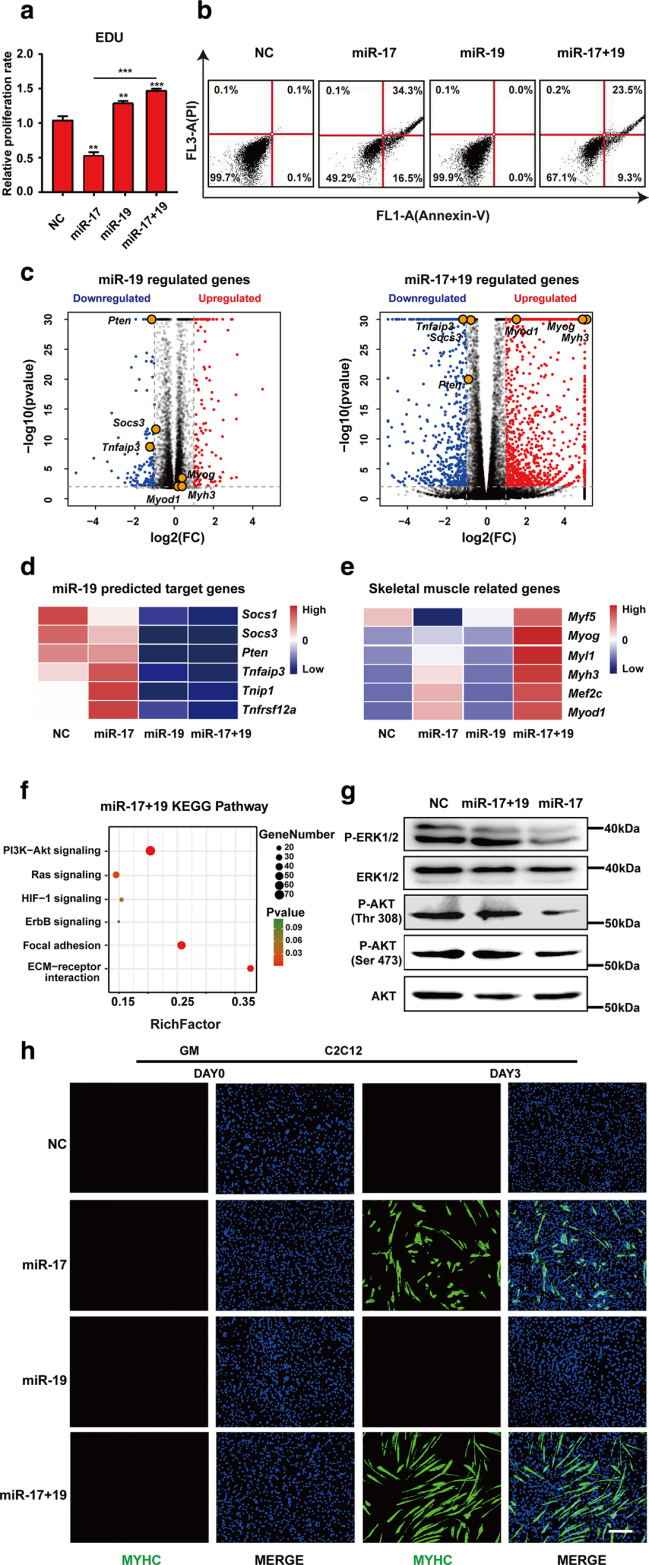
miR-19 complemented miR-17 in promoting muscle differentiation. **a** An EDU incorporation assay. The proliferation rate of C2C12 cells was decreased by miR-17 but was increased by miR-19 (mean ± SEM, **P < 0.01, ***P < 0.001). **b** Apoptosis analyses. C2C12 cells were transfected with miR-17 or miR-17 + 19 for 3 days, and then both the attached cells and the supernatant were harvested and stained with annexin V and PI for subsequent FACS analysis. The addition of miR-19 partially reversed the lethal effects of miR-17. **c** Volcano plots of − log10 (adjusted *P* value) vs. log2 (fold change, FC) of all differential genes after miR-19 or miR-17 + 19 treatment according to the RNA-seq data. The threshold (*P* = 0.05) is indicated with a dashed line. *Pten*, *Socs3* and *Tnfaip3* are highlighted as key downregulated genes, while *Myh3*, *Myod1* and *Myog* are indicated as major myogenic markers. **d** A heatmap of the Targetscan database’s representative predicted targets of miR-19 that were downregulated after miRNA treatment according to the RNA-seq data. **e** A heatmap of the skeletal muscle-related genes that were differentially expressed after miRNA treatment according to the RNA-seq data. **f** KEGG enrichment analysis of the miR-17 + 19 upregulated genes. Notably, PI3K–AKT and Ras–MAPK signalling pathways are recommended. **g** ERK1/2 and AKT (Thr308) were re-activated by miR-19. **h** MYHC immunostaining revealed that miR-17 + 19 promoted C2C12 cell differentiation in GM (growth medium) better than miR-17 alone did (scale bar = 100 μm)

Upon miR-17 treatment, an obvious decrease in cell number, accompanied by an increase in cell debris, was noticed in C2C12 cells. Indeed, 48 h post-GM incubation, miR-17 inhibited cell proliferation, as revealed by EDU (5-ethynyl-2′-deoxyuridine) immunofluorescence staining (Figs. 4a, S4a), and also caused many cells to undergo apoptosis, with 50.8% positive for annexin V and 34.4% positive for PI (propidium iodide) (Figs. 4b, S4b). These effects actually constrain the efficacy of miR-17 in inducing skeletal myogenesis, as myotube formation is dependent on cell fusion. Interestingly, miR-19, another member of the cluster, is capable of promoting cell proliferation and survival. Therefore, we tested whether it could complement miR-17 in this respect.

Indeed, miR-19 increased C2C12 cell proliferation regardless of the presence of miR-17 (Figs. 4a, S4a). Meanwhile, upon co-transfection, it partially reversed the pro-apoptotic role of miR-17 by reducing 18% of annexin V and 10.8% of PI signals (Fig. 4b). A series of genes that can activate inflammation and/or suppress survival, including *Pten*, *Socs3* and *Tnfaip3*, was significantly downregulated in samples treated with miR-19 alone or miR-17 plus miR-19 (miR-17 + 19) (Figs. 4c, d, S4d). These genes were potential targets of miR-19 (Fig. 4d). Notably, *Myf5* reduction by miR-17 was also counteracted by the addition of miR-19 (Fig. 4e). Some genes downregulated by miR-17 but upregulated by miR-17 + 19 were enriched to functions such as cell cycle and cell division; however, the enrichment was not significant (Fig. S4c). Then, we turned to signalling pathways that were upregulated in the miR-17 + 19 group. Since the Ras–MAPK and PI3K–AKT pathways were recommended by KEGG enrichment (Fig. 4f), we measured ERK1/2 and AKT activation by western blotting. Indeed, miR-19 reversed the dephosphorylation effects of miR-17 on ERK1/2, AKT (Thr308) and AKT (Ser473) (Figs. 4g, S4f).

Notably, *Ccnd2*, *Jak1* and *Rhoc* were still significantly downregulated in miR-17 + 19 samples (Fig. S4e). In fact, myogenic differentiation was not antagonised but was facilitated by the supplementation of miR-19, as indicated by the higher expression of *Myh3* (Figs. 4e, S4g). Therefore, the combination of miR-17 and miR-19 was better in promoting C2C12 cell differentiation, which resulted in longer myotubes containing more nuclei, as reflected by MYHC immunostaining (Figs. 4h, S4i), fusion index and differentiation ratio analyses (Fig. S4h, j). By dividing myotubes into the following three categories: 1–4 nuclei (small), 4–10 nuclei (middle) and > 10 nuclei (large), it was revealed that miR-17 + 19 increased both the total number of myotubes and the number of nuclei per myotube (Fig. S4k).

### The healing potential of miR-17 + 19 in injured mouse skeletal muscle

**Fig. 5:**
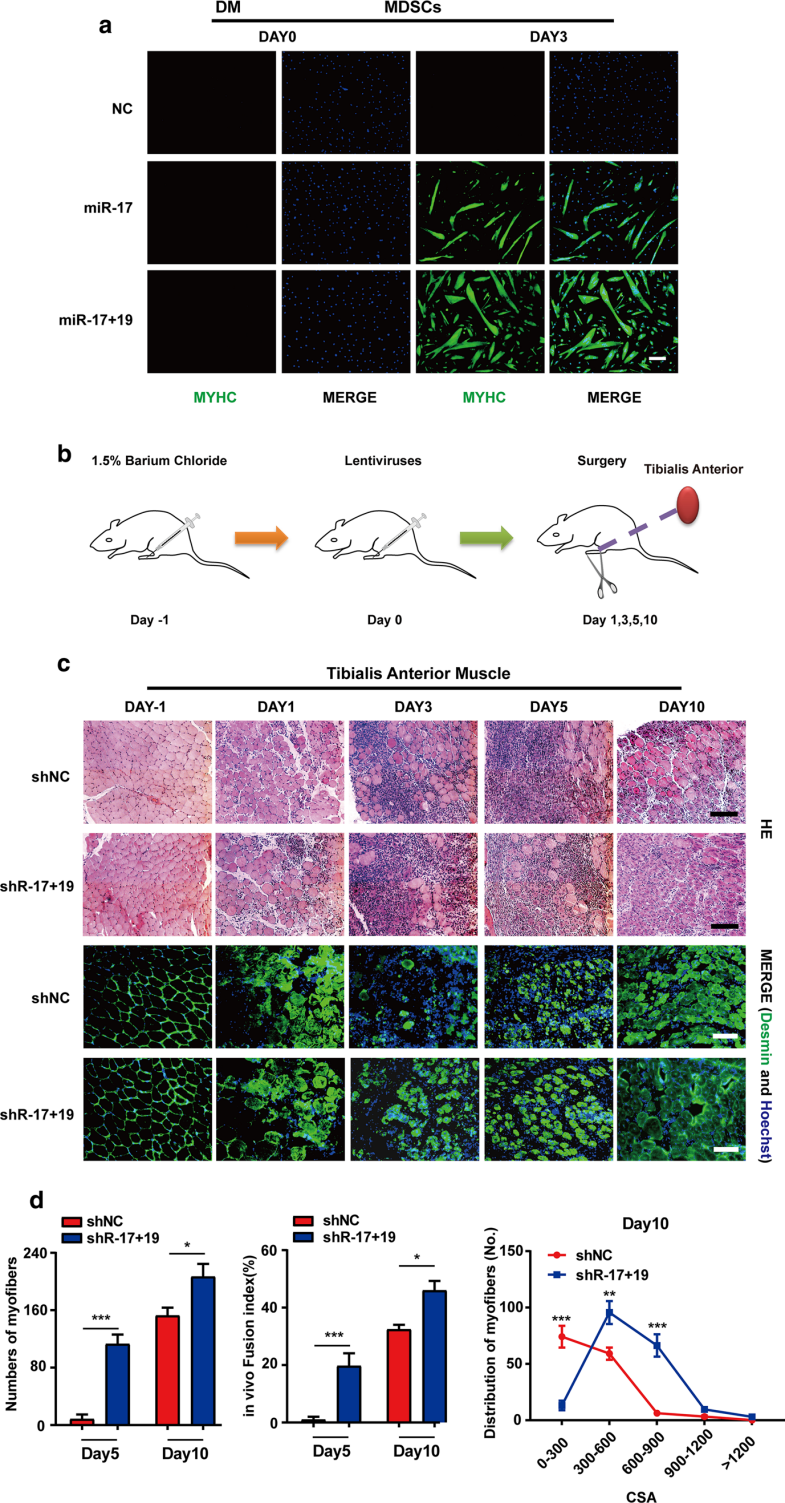
The healing potential of miR-17 and miR-19 in injured mouse skeletal muscles. **a** miR-17 or miR-17 + 19 could promote the differentiation of primary bovine MDSCs (skeletal muscle-derived satellite cells). The cells transfected with the miRNA mimics were cultured for 3 days in DM (differentiation medium) and were then examined by MYHC immunostaining (scale bar = 100 μm). **b** The schematic of the muscle regeneration model. Mouse tibialis anterior muscles were injected with 1.5% BaCl_2_ followed by an injection of lentiviruses expressing shRNAs 24 h later. The lentivirus injection day was set as day 0. **c** Histology analyses of the tibialis anterior muscles from mice injected with shNC or shR-17 + 19. The muscles were collected on the indicated days (scale bar = 100 μm), and their cross sections were examined by H&E and desmin stainings. The immunosignal of desmin indicated that shR-17 + 19 advanced muscle regeneration (scale bar = 100 μm). **d** Shown (left to right) are the number of the newly formed muscle fibres of various CSAs (cross-sectional areas, μM^2^) on day5 and day10, the in vivo fusion index for regenerating myofibres on day5 and day10, and the numbers and distribution of regenerating myofibres on day 10 (mean ± SEM, **P < 0.01, ***P < 0.001)

We then proceeded to test the potential of miR-17 + 19 in muscle damage repair. First, miR-17 and miR-17 + 19 were transfected into primary MDSCs isolated from foetal calves, respectively. MYHC staining showed that both promoted MDSC differentiation (Fig. 5a), and miR-17 + 19 resulted in more myotubes and a higher fusion efficiency than miR-17 alone did (Fig. S5a). Next, we used 1.5% BaCl_2_ (barium chloride) to damage mouse tibialis anterior muscles and injected a mixture of 1% Matrigel and lentiviral vectors containing the shRNAs of miR-17 and miR-19b-1 into the muscles 24 h later (Fig. 5b); this was after the pro-differentiation effects of the shRNAs were validated in C2C12 cells (Fig. S5b, e). The increased expression of miR-17 and miR-19 in the tibialis anterior muscles after injection was confirmed by qPCR (Fig. S5c). Paraffin section analysis showed that the regeneration process was advanced in muscles expressing shRNA-17 plus shRNA-19b-1 (shR-17 + 19) since day 3, with a larger size and counts of newly generated muscle fibres compared to those in the NC muscles. After 10 days, the inflammatory infiltration was significantly reduced, and the regeneration process was almost complete (Figs. 5c, d, S5d). Simultaneously, the immunostaining of desmin, a marker of newly generated muscle fibres [24, 25], confirmed the advanced phenotype in muscles treated with shR-17 + 19 (Fig. 5c).

## Discussion

The miR-17–92 cluster is one of the most investigated miRNA clusters, especially in tumourigenesis [10]. Surprisingly, the reported effects of this cluster on skeletal myogenesis were controversial. As mentioned above, two groups have shown that miR-20a could stimulate the differentiation of both C2C12 [21] and QM-7 cells [22]. However, another group holds an opposite opinion that miR-17, -20a and -92a could block the differentiation of C2C12 cells [23]. Here, we demonstrated that miR-17 and -20a could effectively advance the differentiation of not only C2C12 myoblasts but also primary MDSCs. In contrast, miR-18a might delay C2C12 cell differentiation, as the treatment with its specific inhibitor significantly promoted the myogenic process. In addition, miR-19 and -92a alone exhibited little influence; however, when co-transfected, miR-19 could reverse the lethal effect of miR-17 and could thus facilitate myotube maturation.

Therefore, we were on the side of Liu et al. [21] and Luo et al. [22] to be opposite to Qiu et al. [23]. For such inconsistency, one possible reason might be that the miRNA mimics, the miRNA-expressing viruses and the transfection method used were all different. Given that the amount of each mimic used for transfection and the expression data of the exogenous miRNAs were missing in the article of Qiu et al., it could not be excluded whether there was a significant difference in miRNA levels that might lead to the inconsistency. Moreover, on one hand, the miRNA mimics we used have been improved for their specificity as each passenger strand is inactivated by proprietary chemical modifications so as to avoid being wrongly incorporated into RISC to cause off-target effects [26]. On the other hand, silencing of the two key proteins of RISC suppressed the pro-differentiation abilities of miR-17 and -20a in GM (Fig. S2), which eliminated the off-target effects related to the immune response [26].

An interesting scenario for the members derived from the same miRNA polycistron is to antagonise each other. This has already been observed for the miR-17–92 cluster members; for example, miR-17 can abrogate the erythroleukemia induced by miR-92a [27]. In myogenesis regulation, a notable antagonism occurs between miR-1 and -133. Although the two well-known myomiRs (muscle-specific miRNAs) are clustered and transcribed together, miR-1 stimulates differentiation, whereas miR-133 maintains proliferation [5]. Here, miR-18a emerged to act against miR-17 and -20a in C2C12 cell differentiation, just like it did in Th17 (T helper 17) cell differentiation [28]. Besides, in C2C12 cells, there was one more antagonism arising in the cluster, as miR-19 could mitigate miR-17-induced apoptosis.

Similar to miR-133 [5], miR-18a was upregulated at the early stages to serve as a brake of the normal differentiation of C2C12 cells. In fact, it was also sharply increased during Th17 cell differentiation to inhibit the process [28]. However, contrary to miR-1 [5], miR-17 and -20a were both downregulated during the normal differentiation of C2C12 cells, in spite of their pro-differentiation abilities. Given the miR-17/20a:miR-18a antagonism and the potent pro-apoptosis effect of miR-17 in C2C12 cells, we hypothesised that in the normal myogenic programme, the anti-differentiation ability of miR-18a is preferred, and accordingly, balanced expression of antagonistic members by post-transcriptional regulation results in the decreased levels of miR-17 and -20a due to their strong effects. Notably, during Th17 cell differentiation, although miR-17, -20a and -19b could enhance the process, there was little change in their expression levels [28].

There are a variety of ways for miRNAs to regulate the expression of target genes. The classic one is through the RISC pathway to mediate mRNA degradation or inhibit translation [29, 30].However, in the non-classic manner that is independent of RISC, miRNAs can also do the reverse to promote mRNA stabilisation [31] and/or accelerate translation [32–34]. Here, by knocking down AGO2 and GW182 (two key proteins of RISC) in C2C12 cells, we found that the ability of miR-17 and miR-20a to accelerate the myogenic programme in GM was largely achieved by degrading their target genes via the RISC pathway as *siAgo2* or *siGw182* strongly inhibited their effects (Fig. S2).

Switching from proliferation to differentiation is critical for skeletal myogenesis. Particularly, upon cell cycle exit, most C2C12 cells will naturally enter the myogenic programme. It has been reported that miR-17 can target many cell cycle-related genes, such as *CCND1* (cyclin D1), *MYC* and *E2F1* [35–37]. Notably, the expression level of *Ccnd1* could determine the myogenic fate of C2C12 cells. For example, in GM, unphosphorylated Pitx2 (paired-like homeodomain 2) could stabilise *Ccnd1* mRNA and thus sustain proliferation, whereas in DM, AKT2 could phosphorylate Pitx2 to downregulate *Ccnd1*, and thus, unlock differentiation [38]. Indeed, the *Ccnd1* level was significantly decreased by both miR-17 and -20a (Fig. 2c), indicating its contribution to the pro-differentiation abilities of the two miRNAs, just as it does to miR-206, a positive myomiR in skeletal myogenesis [39].

CCND2 is another key cyclin that controls the G1/S transition of the cell cycle [40]. A previous study indicated that the upregulation of the miR-17–92 cluster could cause significant changes in its expression; however, whether it is the direct target of miR-17 was not verified [41]. Another study reported that *Ccnd2* could be reduced by miR-195 and miR-497, which contributed to the promotion of C2C12 cell differentiation [42]. Indeed, similar to miR-17, *siCcnd2* alone could advance the myogenic programme in the presence of GM (Figs. 3e, S3d). Thus, we demonstrated that *Ccnd2* is also an important contributor to the pro-differentiation ability of miR-17, with its 3′UTR region being directly targeted. Moreover, as the decreased luciferase signal of the *Ccnd2* 3′UTR reporter was not fully rescued by mutating the predicted site, this region might contain at least one other miR-17 site that was ignored by the three databases (TargetScan, MicroRNA and MiRDB).

As key kinases in the JAK–STAT signalling pathway, JAKs play important, although different, roles in myogenesis. JAK1 has a marked effect on myoblast proliferation, whereas JAK2 and JAK3 mainly function in myotube formation [43, 44]. Notably, *siJak1* alone was also enough to advance the myogenic programme in GM (Figs. 3e, S3d); this is consistent with the previous finding that the downregulation of JAK1 switched on the muscle fate of C2C12 cells by directly affecting STAT1 activation [45]. Besides, *JAK1* has already been shown to be the direct target of miR-17 in T-cell survival [46], which was validated here (Fig. 3a, b). Therefore, in parallel to the two cyclins, miR-17 also acts on the pivotal JAK1–STAT1–STAT3 cascade to cease proliferation and thus drive the differentiation of C2C12 cells.

Here, *Rhoc* was verified for the first time to be the target of miR-17 (Fig. 3a, b). However, unlike *siCcnd2* and *siJak1*, *siRhoc* alone could only accelerate differentiation in DM, but not in GM (Fig. 3e), even though it is also involved in tumour cell proliferation or initiation [47]. Nevertheless, as a small (~ 21 kDa) signalling G protein belonging to the Rac subfamily, RHOC is also important in actin cytoskeleton organisation, cell shape formation and cell motility regulation [48, 49]. In the late stage of muscle differentiation, ECM (extracellular matrix) is required for cell fusion. However, RHOC can lead to ECM degradation, and finally, to cell polarity loss [50]. Moreover, RHOC can interact with ROCK1 (Rho-associated coiled-coil containing protein kinase 1) to phosphorylate and activate CNN3 (calponin 3), a negative regulator of muscle differentiation and cell fusion [51–53]. Altogether, the downregulation of RHOC might facilitate myotube formation via increased ECM expression and decreased CNN3 activation [36, 52, 54].

Although miR-17 emerges as a potent inducer of skeletal myogenesis via targeting a diverse set of genes, its pro-differentiation ability is largely hampered by the concomitant lethal effect, as the cell number required for myoblast fusion is reduced. Interestingly, this deficiency can be complemented by miR-19 (Figs. 4, S4), another miR-17–92 cluster member that is known to be capable of promoting cell proliferation and survival [55, 56]. This might be largely attributed to its targets that were significantly downregulated in the miR-17 + 19 samples, such as PTEN, SOCS3 and TNFAIP3 (Figs. 4e, S4d), which are involved in the PI3K–AKT signalling pathway, skeletal muscle insulin resistance and TNF-mediated apoptosis, respectively [57–60]. Indeed, the AKT pathway was re-activated by miR-17 + 19, which was suggested by KEGG analysis and confirmed by western blotting, so was the MAPK pathway that is also significant in modulating cell proliferation and survival (Fig. 4g, h) [61]. In other words, miR-19 could reverse the lethal effect of miR-17 on C2C12 cells via, at least in part, the re-activation of the two pathways. Consequently, the combination of miR-17 and -19 exhibited a better effect on myogenic differentiation, which also effectively improved in vivo muscle regeneration after injury. Our findings thus offer new insights into the mechanisms of muscle differentiation and a potential strategy for meat production increase and skeletal muscle disease therapy.

## Materials and methods

### Cell culture

C2C12 myoblasts and 293T cells were purchased from American Type Culture Collection (ATCC, USA). MDSCs were isolated as previously described [62]. In a humidified incubator with 5% CO_2_ at 37 °C, C2C12 and 293T cells were grown in DMEM (HyClone, UT) with 10% foetal bovine serum (Gibco, USA) and 1% GlutaMAX Supplement (Gibco), while MDSCs were cultured in DMEM with 15% foetal bovine serum, 10% heat-inactivated horse serum (Gibco) and 1% GlutaMAX Supplement. For the induction of differentiation, both cells were switched to DMEM with 2% heat-inactivated horse serum.

### RNA oligonucleotides and transfection

The miRNA mimics and inhibitors were purchased from Invitrogen (USA) and GenePharma (China), respectively. All siRNAs were purchased from GenePharma, and their sequences are listed in Table S1. C2C12 cells and MDSCs were seeded into 24-well plates and were transfected with 50 nM RNA oligonucleotides using Lipofectamine™ RNAiMAX (Invitrogen) according to the manufacturer’s instructions.

### Immunofluorescence staining

For staining, the cells cultured in 24-well plates were fixed with 4% PFA (paraformaldehyde) for 20 min and were then permeabilized with 0.3% Triton X-100 for 17 min at RT (room temperature). After the blocking with 10% horse serum for 50 min at 37 °C, the cells were incubated overnight at 4 °C with a MYHC antibody (1:200, cat. #bs-5885R, Bioss Antibodies, China). Then, the secondary antibody Alexa Fluor 488 (1:200, cat. #A0423, Beyotime, China) was incubated with the samples for 1 h at 37 °C. The cell nuclei were stained by DAPI (1:1000, cat. #C1002, Beyotime) for 5 min at RT. The MYHC and DAPI signals were visualised by a DMI3000B fluorescence microscope (Leica, Germany). The fusion index was determined by measuring the fraction of nuclei contained within the fused myoblasts with ImageJ software. EDU staining was performed according to the manufacturer’s protocol using the Cell-Light™ EdUTP Apollo^®^567 TUNEL In Situ Detection Kit (20T) (cat. #C10810-1, Ribobio, China).

### RNA isolation and qRT-PCR

The total RNA was extracted using the TRIzol RNA isolation system (Takara, China) according to the manufacturer’s instructions. Real-time PCR was performed using SYBR Green Master (Cat#04913914001, Roche, Switzerland) in a Light-Cycler 480 System (Roche). The following conditions were used: 95 °C for 30 s followed by 40 amplification cycles at 95 °C for 10 s and 60 °C for 15 s. The primers used are listed in Table S2. The relative gene expression levels were detected 48 h after culturing in GM conditions and quantified by normalisation to those of endogenous β-actin using the 2^−ΔΔCt^ method. The relative miRNAs expression levels were quantified by normalisation to those of endogenous U6 using the 2^−ΔΔCt^ method as well.

### RNA sequencing and bioinformatics analysis

C2C12 myoblasts were seeded into six-well plates and were then treated with miRNA mimics for 48 h. The total RNA samples from three biological replicates were sent to Novogene (China) for RNA-seq (Accession number: PRJNA534274). The library preparations were sequenced on an Illumina HiSeq platform, and 125-bp/150-bp paired-end reads were generated. Hisat2 was selected as the mapping tool. The DESeq2 R package (1.16.1) was used as the differential expression analysis tool. Genes with an adjusted *P* value < 0.05 and |log2 (FoldChange)| > 1 found by DESeq2 were assigned as differentially expressed. The RNA-seq data were analysed using DAVID Bioinformatics Resources 6.8. Target prediction was performed using the three following databases: TargetScan, MicroRNA and MiRDB.

### Western blot analysis

C2C12 cell lysates were prepared using RIPA buffer containing protease inhibitors, phosphatase inhibitors and dithiothreitol. The protein concentration was measured using the BCA Kit (Beyotime). Western blot analyses were performed with the following primary antibodies: anti-AKT (cat. #4691), anti-p-AKT 473 (cat. #4060), anti-p-AKT 308 (cat. #2965), anti-ERK1/2 (cat. #9102), anti-p-ERK1/2 (cat. #9101), anti-CCND2 (cat. #3741) and anti-JAK1 (cat. #3344) from Cell Signalling Technology (USA) (all at 1:1000), anti-RHOC (1:500, cat. #D260057) from Sangon Biotech (China), and anti-β-actin (1:5000, cat. #A5441) from Sigma-Aldrich (USA). After an overnight incubation at 4 °C, the membranes were incubated for 1 h at RT with the following secondary antibodies: anti-rabbit or anti-mouse IgG-horseradish peroxidase (HRP) (Proteintech, USA). Finally, the protein bands were detected using the Super ECL Detection Reagent (Tanon, China).

### Flow cytometry analysis of cell apoptosis

After 48 h of treatment with miR-17 or miR-17 + 19, cells were subjected to double immunostaining of annexin V-FITC and PI according to the protocol of the Apoptosis Detection Kit (Vazyme Biotech, China). The samples were detected using a BD Accuri™ C6 Plus Flow Cytometer (BD Biosciences, USA) and were analysed by FlowJo X software. For the visualisation of annexin V signals, Delta Vision OMX was used to display a higher resolution of the apoptotic phenotype.

### Plasmid construction

The 3′UTR fragments of *Ccnd2*, *Jak1* and *Rhoc* containing putative miR-17-binding sites were amplified by PCR. The corresponding mutagenesis of the binding sites was achieved by overlapping PCR. Afterwards, the PCR products were inserted into the pMIR-report vector (Promega, USA). All constructs were confirmed by DNA sequencing.

### Dual-luciferase assay

For the dual-luciferase assay, 293T cells were co-transfected with each pMIR-Report-3′UTR construct, control Renilla pRL-SV40 (Promega), and either miR-17 or miRNA NC using Lipofectamine™ 2000 (Invitrogen). After 48 h, the cells were analysed using the Dual-Luciferase Reporter Assay System Kit (Promega) on a GloMaxTM 20/20 Luminometer (Promega).

### Lentivirus packaging

For lentivirus packaging, using the polyethylenimine-based transfection method [63], 293T cells were transfected with the purified viral plasmids expressing shRNA-17 and shRNA-19b-1, which were purchased from GenePharma. The serum was added 6–8 h after transfection, and the virus-containing supernatant was collected after 72 h. The viruses were then concentrated by ultracentrifugation to at least 10^10^ pfu/ml.

### Mouse muscle regeneration model and lentivirus injection

C57BL/6 female mice were purchased from the Second Affiliated Hospital of Harbin Medical University (China). The mice used were at 8 weeks of age and had similar weights. A mouse muscle injury model was achieved by injecting 50 μl 1.5% BaCl_2_ per leg into the tibialis anterior muscle. After 24 h, 50 μl of lentiviruses expressing shRNA-17 and shRNA-19b-1 was injected. Two tibialis anterior muscles from each mouse were collected at day 0, day 1, day 3, day 5 and day 10 after the lentivirus injection. Each group contained 12 mice, 6 of which were used for shRNA expression analyses, and the other 6 were used for histology analyses. All animal procedures were approved by the Animal Care and Use Committee of Northeast Forestry University, and were carried out in accordance with the guidelines.

### Histology

The skeletal muscle tissues were embedded in paraffin, cut into 5-μm thick sections, and stained with H&E (haematoxylin and eosin, cat. #C0105, Beyotime) or a desmin antibody (1:200, cat. #AF0132, Beyotime, China). The regenerating areas were captured by a DMI3000B fluorescence microscope (Leica).

### Statistical analysis

The results from at least three independent experiments were analysed by Student’s *t* test, one-way ANOVA or two-way ANOVA.

## Electronic supplementary material

Below is the link to the electronic supplementary material.

Supplementary material 1 (DOCX 2864 kb)

Supplementary material 2 (DOCX 33 kb)
