# Supplementary material for: MiR-17 and miR-19 cooperatively promote skeletal muscle cell differentiation
Source: Cell Mol Life Sci. 2019 Jun 18;76(24):5041–54. doi: 10.1007/s00018-019-03165-7 (PMC6881278; doi:10.1007/s00018-019-03165-7)
Supplement: Supplementary file 1 — Supplementary material 1 (DOCX 2864 kb) [file 18_2019_3165_MOESM1_ESM.docx]

**Supplement Figure 1**

**
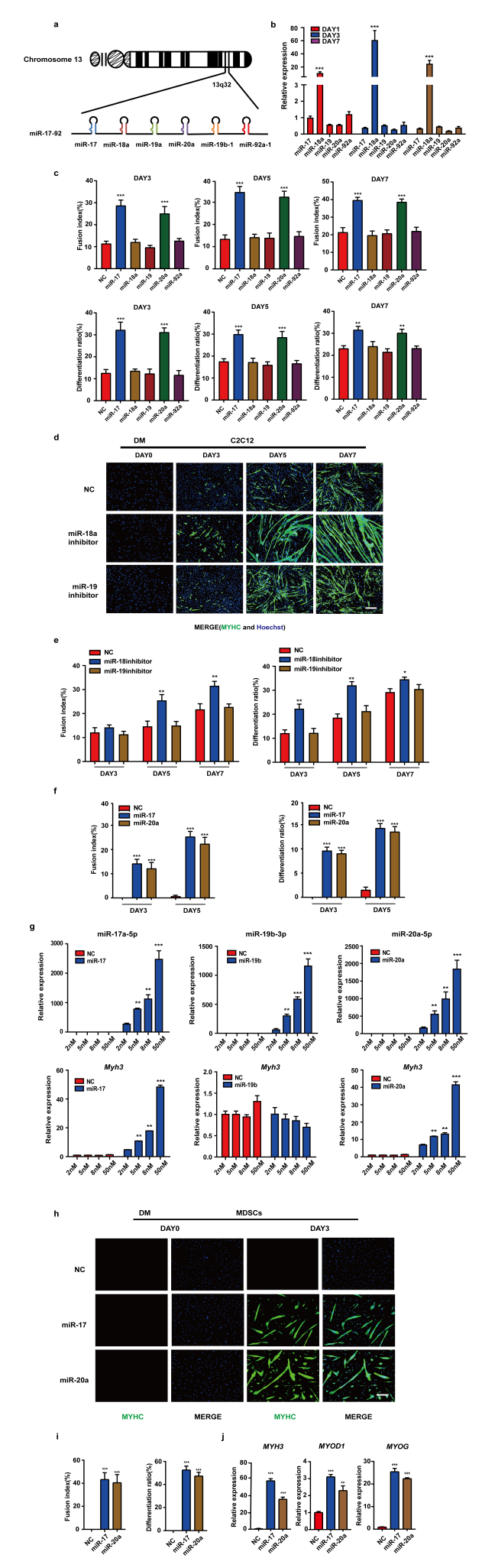
**

**Supplement Figure 2**

**
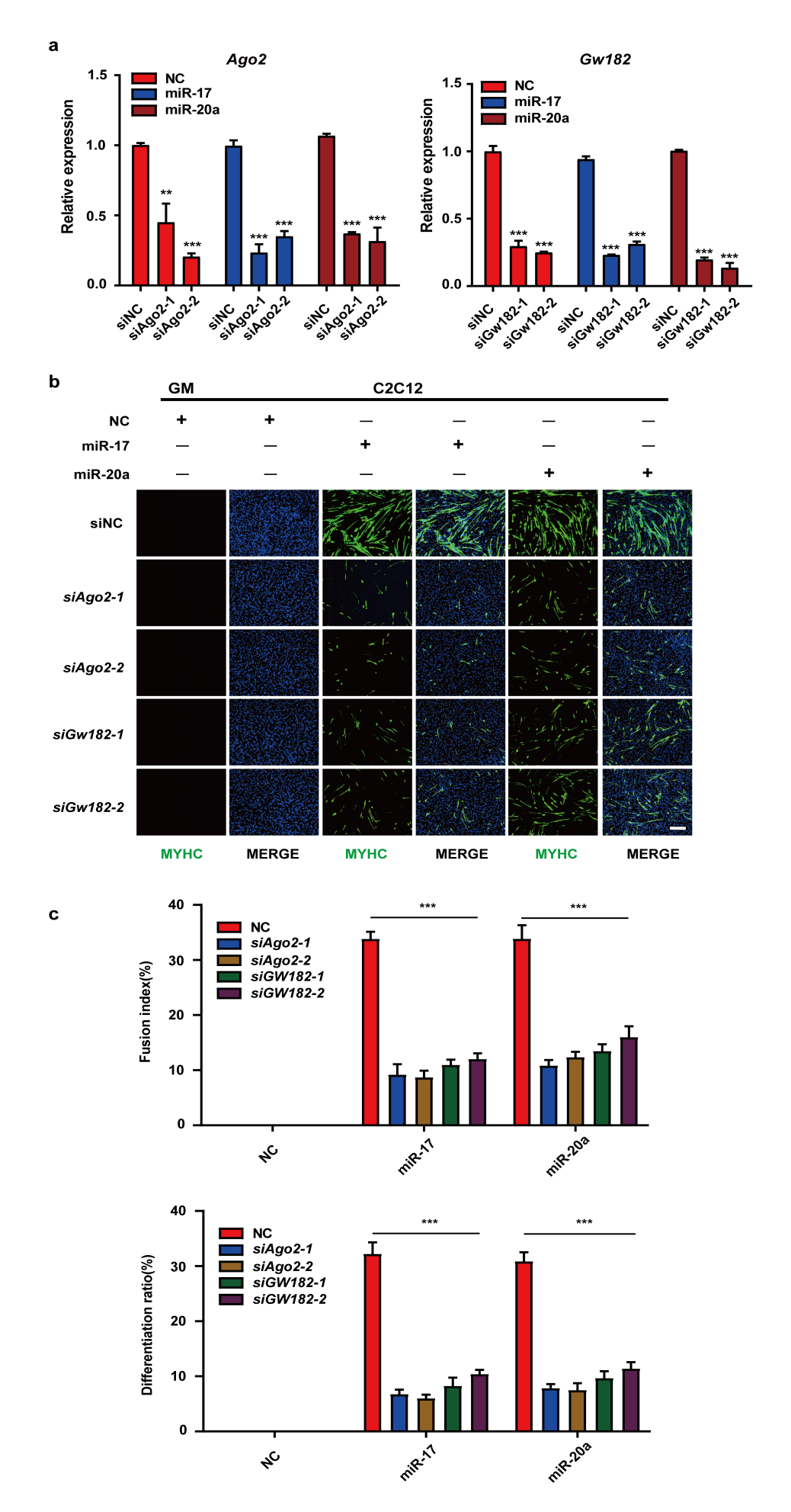
**

**Supplement Figure 3**

**
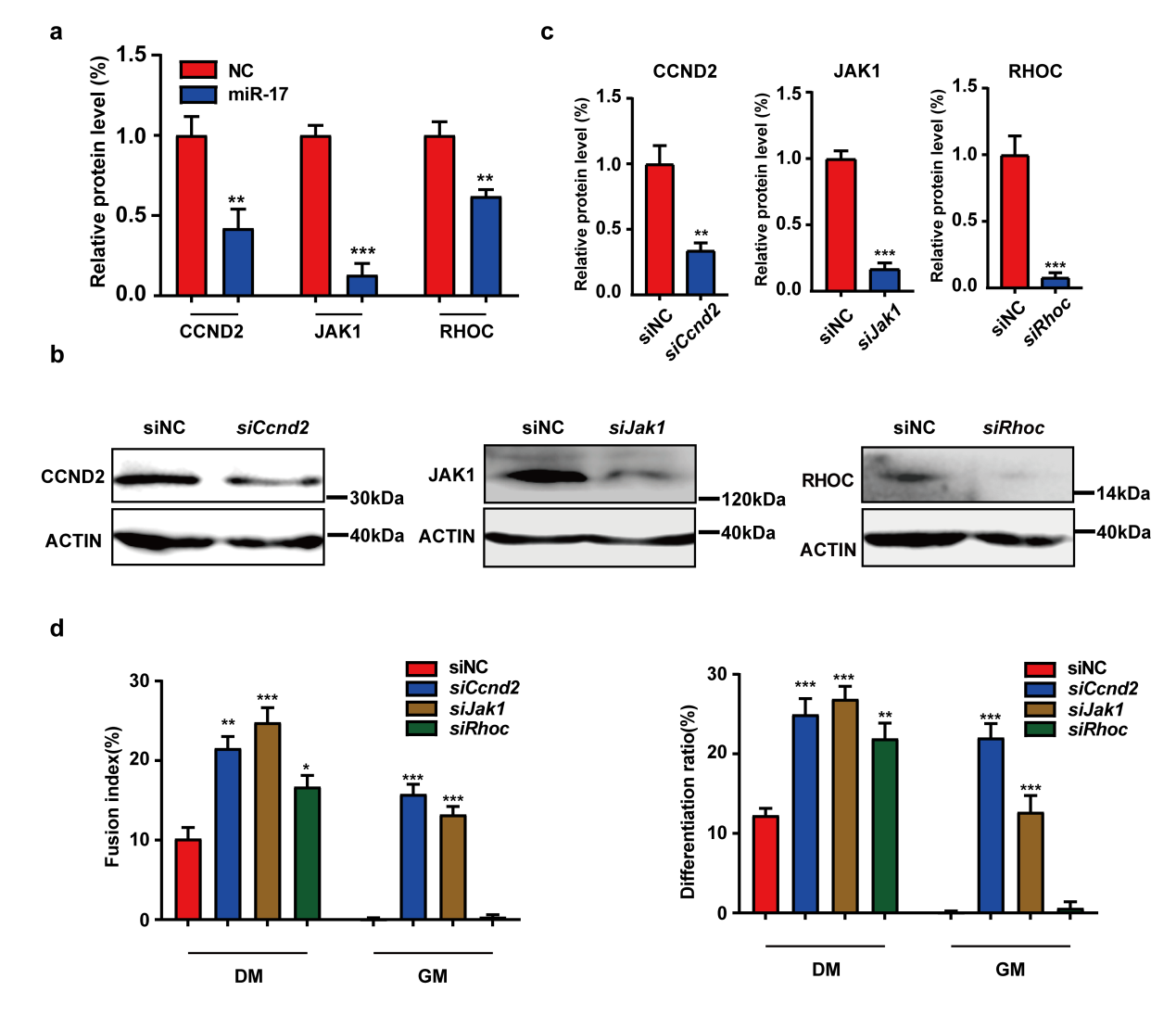
**

**Supplement Figure 4**

**
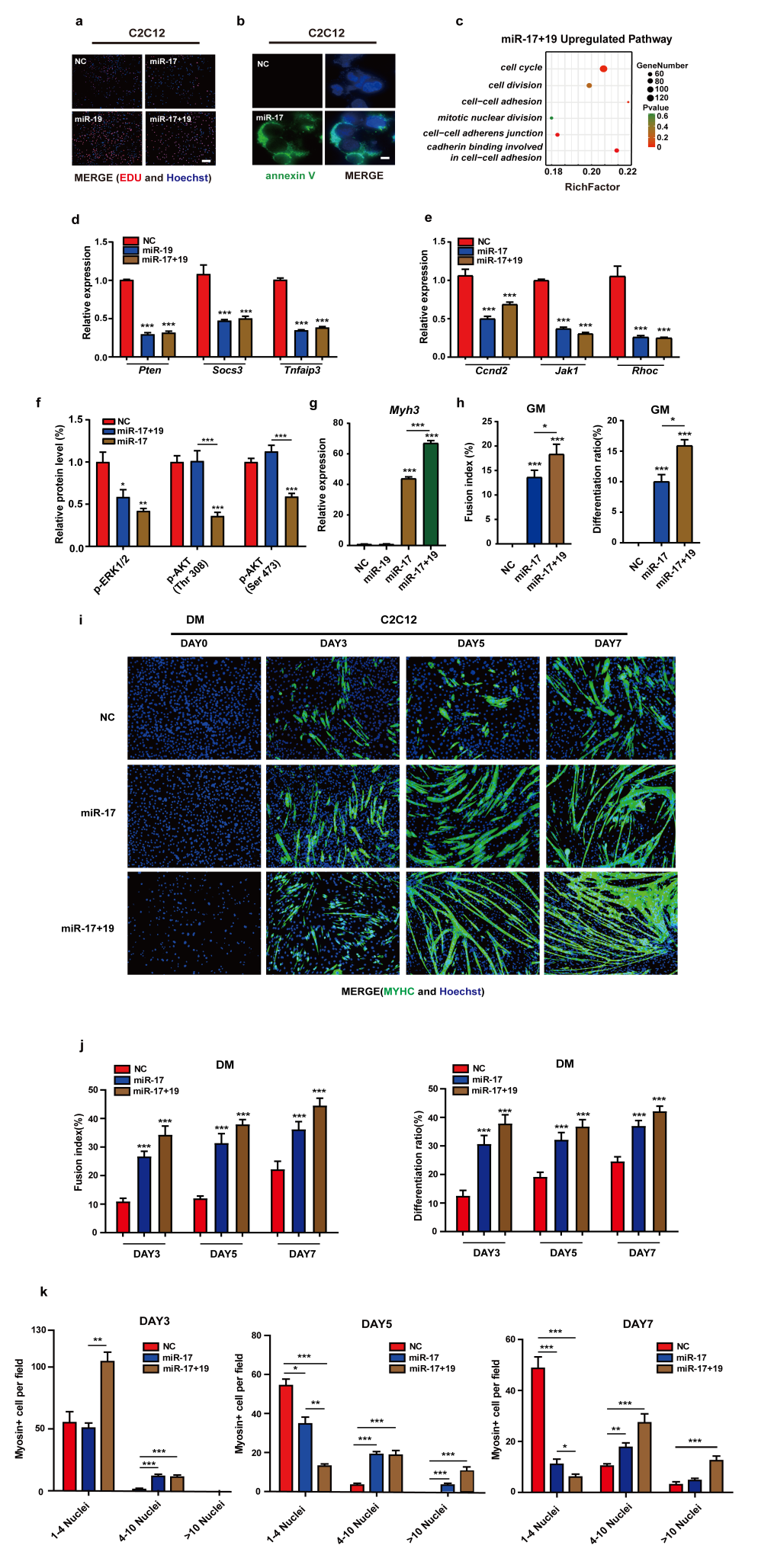
**

**Supplement Figure5**

**
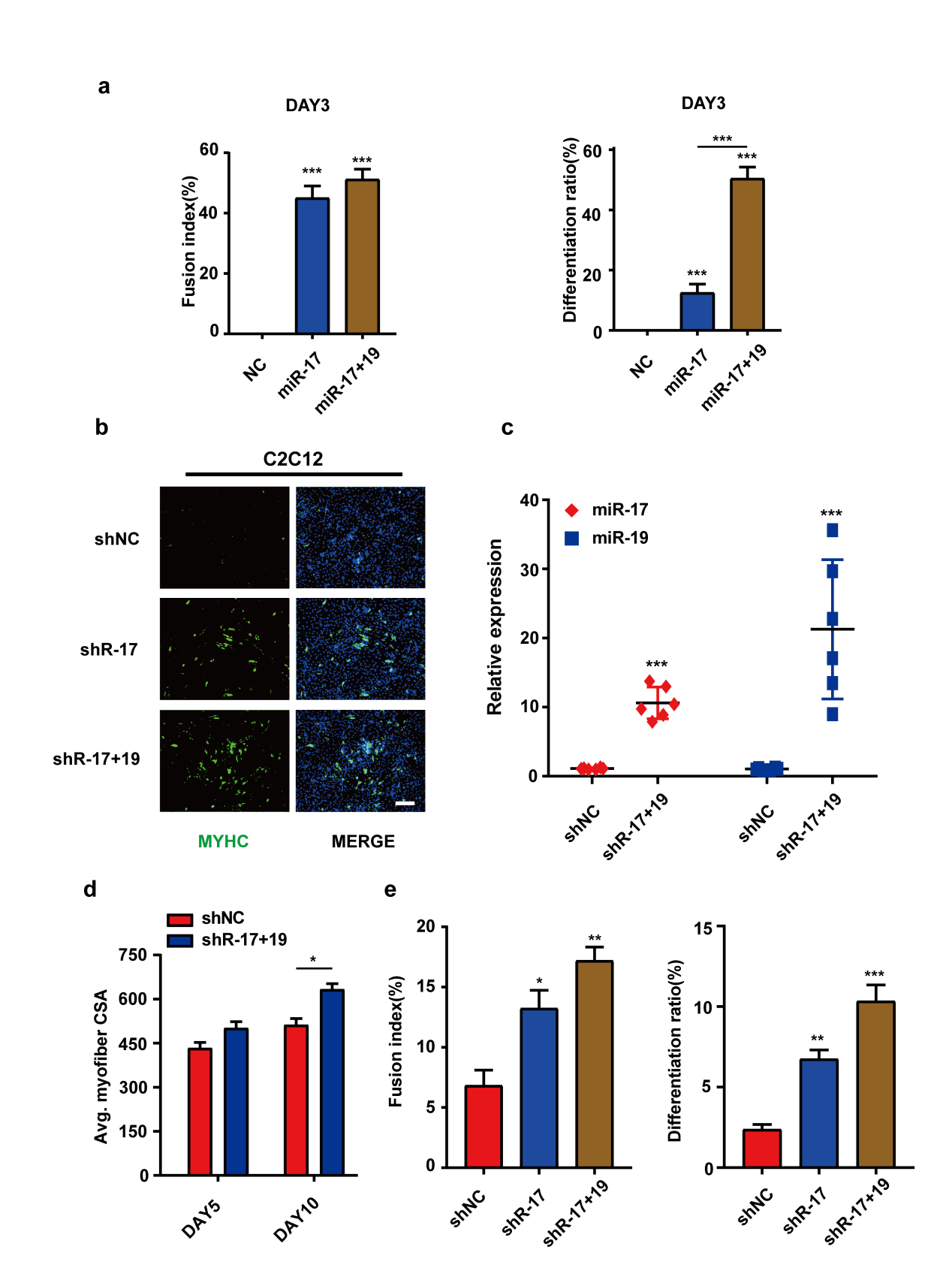
**

**Supplementary figure legends**

**Fig S1 Different roles of the miR-17-92 cluster members in muscle differentiation.** (**a**) A schematic representation of the miR-17-92 cluster on human chromosome 13. The cluster consists of six miRNAs: miR-17, -18a, -19a, -20a, -19b-1, and -92a-1. (**b**) The endogenous expression patterns of each miR-17-92 cluster member detected on days 1, 3 and 7 after DM (differentiation medium) induction. The relative (miRNA/U6) level of miR-17 on day 1 was set to 1.0 (mean + SEM, ***P<0.001). (**c**) Fusion index and differentiation ratio analyses of the mimic-treated cells in DM on days 3, 5 and 7 (***P<0.001, n=6). (**d**) MYHC immunostaining of C2C12 cells after miRNA inhibitor treatment. The inhibitor of miR-18a, but not that of miR-19, promoted myogenic differentiation in DM (scale bar=100 μm). (**e**) Fusion index and differentiation ratio analyses of the inhibitor-treated cells in DM on days 3, 5 and 7 (**P<0.01, n=6). (**f**) Fusion index and differentiation ratio analyses of C2C12 cells treated with the miR-17 or -20a mimic in GM (growth medium) on days 3 and 5 (***P<0.001, n=6). (**g**) The expression levels of the miRNAs and *Myh3* detected by qRT-PCR 48 h post mimic transfection. C2C12 cells were transfected with different concentrations of the miR-17, -19 and -20a mimics. *Myh3* was upregulated by miR-17 and -20a but not by miR-19. The levels were normalized as *miRNA/U6* and *Myh3*/*Gapdh*. The relative levels of the NC (negative control) at 2 nM were set to 1.0 (mean + SEM, **P<0.01, ***P<0.001). (**h**) MYHC immunostaining of primary bovine MDSCs (skeletal muscle-derived satellite cells) treated with the miR-17 or -20a mimic. The differentiation process were promoted in DM (scale bar=100 μm). (**i**) Fusion index and differentiation ratio analyses of the mimic-treated MDSCs (***P<0.001, n=6). (**j**) The increased expression of *MYH3*, *MYOD1* and *MYOG* in MDSCs treated with the miR-17 or -20a mimic for 48 h. The relative (*mRNA*/*GAPDH*) levels of the NC were set to 1.0 (mean + SEM, **P<0.01, ***P<0.001).

**Fig S2 miR-17 and miR-20 promoted C2C12 cell differentiation via the classical RISC pathway.** (**a**) The interference efficiency test of the siRNAs of *Ago2* and *Gw182*. At 48 h post transfection, the mRNA levels of *Ago2* and *Gw182* were significantly down-regulated by their siRNAs, respectively, as revealed by qRT-PCR. The efficiency was not affected by the presence of the miRNA mimics. The relative (*mRNA/Gapdh*) levels of the NC (negative control) and siNC group were set to 1.0 (mean + SEM, **P<0.01, ***P<0.001). (**b**) MYHC immunostaining of C2C12 cells co-transfected with the indicated combinations of mimic and siRNA for 3 days in GM (growth medium). Silencing of the two key components of RISC (RNA-induced silencing complex) suppressed the pro-differentiation effects of miR-17 and -20a (scale bar=100 μm). (**c**) Fusion index and differentiation ratio analyses of the mimic and siRNA-treated cells. (***P<0.001, n=6).

**Fig S3 *Ccnd2*, *Jak1* and *Rhoc* were directly targeted by miR-17 in promoting C2C12 cell differentiation.** (**a**) The relative protein levels of CCND2, JAK1 and RHOC after miR-17 treatment were quantified based on three independent experiments (mean + SEM, **P<0.01, ***P<0.001). (**b**) The interference efficiency test of the siRNAs of *Ccnd2*, J*ak1* and *Rhoc*. After 48 h of treatment, the protein levels of CCND2, JAK1 and RHOC were reduced by their siRNAs, respectively, as revealed by western blotting. ACTIN was used as the internal control. (**c**) The relative protein levels of CCND2, JAK1 and RHOC after siRNA treatment were quantified based on three independent experiments (mean + SEM, **P<0.01, ***P<0.001). (**d**) Fusion index and differentiation ratio analyses of the siRNA-treated cells in either DM (differentiation medium) or GM (growth medium) (***P<0.001, n=6).

**Fig S4 miR-19 complemented miR-17 in promoting muscle differentiation.** (**a**) The staining of EDU (scale bar=100 μm). (**b**) The staining of annexin V (scale bar=16.67 μm). (**c**) KEGG enrichment of the genes that were downregulated by miR-17 but upregulated by miR-17+19. (**d**) The mRNA levels of *Pten*, *Socs3* and *Tnfaip3* were decreased by miR-19 or miR-17+19, as revealed by qRT-PCR after 48h cultured in GM. The relative (*mRNA*/*Gapdh*) levels of the NC (negative control) were set to 1.0 (mean + SEM, **P<0.01, ***P<0.001). (**e**) *Ccnd2*, *Jak1* and *Rhoc* were still downregulated in miR-17+19 samples. (**f**) The relative protein levels of p-ERK and p-AKT were quantified based on three independent experiments (mean + SEM, *P<0.05, **P<0.01, ***P<0.001) (**g**) The mRNA level of *Myh3* was higher in the miR-17+19 group than that in the miR-17 group. (**h**) Fusion index and differentiation ratio analyses of C2C12 cells transfected with miR-17 or miR-17+19 in GM (growth medium) on day 3 (mean + SEM, **P<0.01, ***P<0.001). (**i**) MYHC immunostaining of C2C12 cells revealed that miR-17+19 outperformed miR-17 in DM (differentiation medium) (scale bar=100 μm). (**j**) Fusion index and differentiation ratio analyses of C2C12 cells transfected with miR-17 or miR-17+19 in DM (mean + SEM, *P<0.05, ***P<0.001). (**k**) Myotube category analysis of C2C12 cells transfected with miR-17 or miR-17+19 in DM (mean + SEM, *P<0.05, **P<0.01, ***P<0.001).

**Fig S5 The healing potential of miR-17 and miR-19 in injured mouse skeletal muscles.** (**a**) Fusion index and differentiation ratio analyses of primary bovine MDSCs (skeletal muscle-derived satellite cells) treated with miR-17 or miR-17+19. (**b**) A functional test of lentiviral shRNA-17 and shRNA-19b-1 in C2C12 myoblasts. MYHC immunostaining was performed 3 days post infection (scale bar=100 μm). (**c**) The overexpression efficiency test of lentiviral shRNA-17 and shRNA-19b-1 in mouse tibialis anterior muscles. The muscles were collected 3 days post lentivirus injection and the levels of mature miRNAs were determined by qRT-PCR. (mean + SEM, ***P<0.001, n=6). (**d**) The average myofibre CSA (cross-sectional area, μM^2^) examined on the indicated days. (**e**) Fusion index and differentiation ratio analyses of C2C12 cells infected with the lentiviruses (*P<0.05, **P<0.01, ***P<0.001, n=6).
