# Supplementary material for: MiR-17 and miR-19 cooperatively promote skeletal muscle cell differentiation
Source: Cell Mol Life Sci. 2019 Jun 18;76(24):5041–54. doi: 10.1007/s00018-019-03165-7 (PMC6881278; doi:10.1007/s00018-019-03165-7)
Supplement: Supplementary file 2 — Supplementary material 2 (DOCX 33 kb) [file 18_2019_3165_MOESM2_ESM.docx]

**Supplement table 1**

**Small interfering RNAs for RNA interference**

siRNA Name Sequence of siRNAs

mmu-siAGO2-1 CGGGAGAACAAUCAAACUATT

UAGUUUGAUUGUUCUCCCGTT

mmu-siAGO2-2 CCCACUGAGUUUGACUUCUTT

AGAAGUCAAACUCAGUGGGTT

mmu-siGW182-1 GAAAUGCUCUGGUCCGCUAUU

UAGCGGACCAGAGCAUUUCUU

mmu-siGW182-2 CUAAUUACUCUGGCGACAAAU

AUUUGUCGCCAGAGUAAUUAG

mmu-siCCND2 CUGCAACAUGGGAACGAAUUA

AUUUGUCGCCAGAGUAAUUAG

mmu-siRHOC GAAACUGAUCUUUGCUGAAGA

UCUUCAGCAAAGAUCAGUUUC

mmu-siJAK1 UCUUACCAGGAUGCGAAUAAA

UUUAUUCGCAUCCUGGUAAGA

**Supplement table 2**

**Primers for quantitative RT-PCR.**

Primer Name Sequences of Primers

mmu-miR-17-Forward TGGGCAAAGTGCTTACAGTG

mmu-miR-17-Reverse CAGTGCGTGTCGTGGAGT

mmu-miR-18a-Forward GGGGTAAGGTGCATCTAGTG

mmu-miR-18a-Reverse CAGTGCGTGTCGTGGAGT

mmu-miR-19-Forward TGGTGTGCAAATCCATGC

mmu-miR-19-Reverse CAGTGCGTGTCGTGGAGT

mmu-miR-20a-Forward TCGGGTAAAGTGCTTATAGTGC

mmu-miR-20a-Reverse CAGTGCGTGTCGTGGAGT

mmu-miR-92a-Forward CCGTATTGCACTTGTCCC

mmu-miR-92a-Reverse CAGTGCGTGTCGTGGAGT

mmu-CCND2-3’UTR-F CCTATGAGCTCCCTCACTAGTCCGAAGAATGCTGGGATGAG

mmu-CCND2-3’UTR-R TGATGAAAGCTGCGCACTAGTGGCCATGACCAAAACGCA

mmu-CCND2-MUT-F CCCCAAAATTTTCCCTCACGTTTTCTAATGGAATGGTTTATAACAAAG

mmu-CCND2-MUT-R CGTGAGGGAAAATTTTGGGGGAGGG

mmu-RHOC-3’UTR-F CCTATGAGCTCCCTCACTAGTTCACGGTGCAACCTTTGACC

mmu-RHOC-3’UTR-R TGATGAAAGCTGCGCACTAGTCTTCCCCAAAGCTTCCTCAAC

mmu-RHOC-MUT-F CACGTGGTGTGTAATATGGGGAAGGC

mmu-RHOC-MUT-R CCCATATTACACACCACGTGAGGATATATTTTTGGCTCAC

mmu-JAK1-3’UTR-F CCTATGAGCTCCCTCACTAGTCAAGACAGAAATGCAAATCAAGTGA

mmu-JAK1-3’UTR-R TGATGAAAGCTGCGCACTAGTTCATGACCAAGAGTCTGGCAAA

mmu-JAK1-MUT-F AACCCTCACGATATAGAGCGAGACACAGGTTTGACG

mmu-JAK1-MUT-R CGCTCTATATCGTGAGGGTTTACTCTTTATACAATAAAC

mmu-CCND2-Forward GGAACTGGTAGTGTTGGGTAAG

mmu-CCND2-Reverse GGGTACATGGCAAACTTGAAG

mmu-RHOC-Forward AGCAAAGATCAGTTTCCAGAGG

mmu-RHOC-Reverse AGGGCTGTCAATGGAGAAAC

mmu-JAK1-Forward CATATAGTGTACCTCTACGGCG

mmu-JAK1-Reverse GTTTGGCAACCTTGAACTTCC

mmu-AGO2-Forward TGCCATGGTACGAGAGTTGCTCAT

mmu-AGO2-Reverse ACGATGAACGTGATTCCTGGCTGA

mmu-GW182-Forward ACTCCTGGCAGTGTCATAAAC

mmu-GW182-Reverse TGTAGTTGGAGGCACGAATG

mmu-MYH3-Forward AAGCTCGTCACTTTGGTACAG

mmu-MYH3-Reverse CACCTCTTTGATTTTGGCTTCC

mmu-MYOG-Forward ACAATCTGCACTCCCTTACG

mmu-MYOG-Reverse GTGATGGCTTTTGACACCAAC

mmu-MYOD1-Forward CCAATGCGATTTATCAGGTGC

mmu-MYOD1-Reverse CGAAAGGACAGTTGGGAAGAG

Primer Name Sequences of Primers

bta-MYH3-Forward TGCTCATCTCACCAAGTTCC

bta-MYH3-Reverse GGCTCACTCTTCACTCTCATG

bta-MYOG-Forward CAGGGAGATAAAGCAAGGGAC

bta-MYOG-Reverse TTTCAGGGAGTGGATTTGGAG

bta-MYOD1-Forward AACCCCAACCCGATTTACC

bta-MYOD1-Reverse CAACAGTTCCTTCGCCTCTC

mmu-PTEN-Forward GAGACATTATGACACCGCCA

mmu-PTEN-Reverse GCACAAATCATTACACCAGTCC

mmu-SOCS3-Forward CCTATGAGAAAGTGACCCAGC

mmu-SOCS3-Reverse TTTGTGCTTGTGCCATGTG

mmu-TNFAIP3-Forward ACAGGACTTTGCTACGACAC

mmu-TNFAIP3-Reverse CTTCTGAGGATGTTGCTGAGG

mmu-GAPDH-Forward CACGGCAAATTCAACGGCACAGTCAAGG

mmu-GAPDH-Reverse GTTCACACCCATCACAAACATGG
